# Supplementary material for: Comparing Fungal Sensitivity to Isothiocyanate Products on Different Botrytis spp
Source: Plants (Basel). 2024 Mar 7;13(6):756. doi: 10.3390/plants13060756 (PMC10974099; doi:10.3390/plants13060756)
Supplement: Supplementary file 1 [file plants-13-00756-s001.zip › Supplementaty material.pdf]

Supplementary Figure S1. Alignment of *BcmfsG* coding sequence and homologous gene sequences from different *Botrytis* species: *B. cinerea* B05.10, *B. pseudocinerea* BP362, *B. medusae* B555, *B. fragariae* BCB16 and *B. byssoidea* MUCL94. Gene sequences were selected after running similarity search by Blastn using BcmfsG as query sequence. Multiple alignments of protein sequences were carried out with Clustal-Omega and were visualized using ESPript 3.0. Blue and green box represent conserved regions between *Botrytis* species within the region corresponding to the domain MFS-1 (pfam07690; interval 136-1140), targets for the design of primers for the identification of *mfsG* gene in other *Botrytis* species by PCR. The arrows indicate the orientation of the primers used for the *mfsG* gene detection.

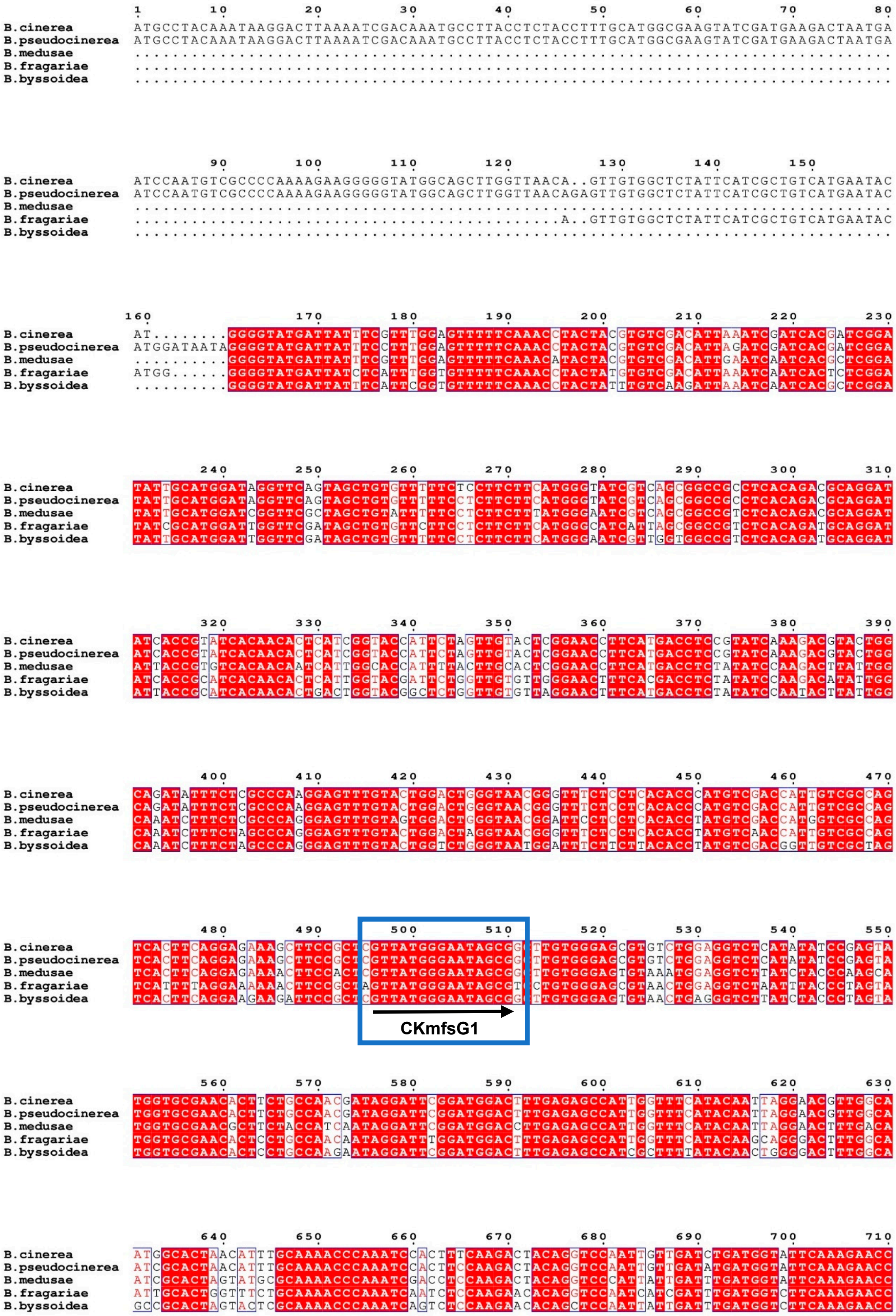

Supplementary Figure S1 (part 2). Alignment of *BcmfsG* coding sequence and homologous gene sequences from different *Botrytis* species: *B. cinerea* B05.10, *B. pseudocinerea* BP362, *B. medusae* B555, *B. fragariae* BCB16 and *B. byssoidea* MUCL94. Gene sequences were selected after running similarity search by Blastn using BcmfsG as query sequence. Multiple alignments of protein sequences were carried out with Clustal-Omega and were visualized using ESPript 3.0. Blue and green box represent conserved regions between *Botrytis* species within the region corresponding to the domain MFS-1 (pfam07690; interval 136-1140), targets for the design of primers for the identification of *mfsG* gene in other *Botrytis* species by PCR. The arrows indicate the orientation of the primers used for the *mfsG* gene detection.

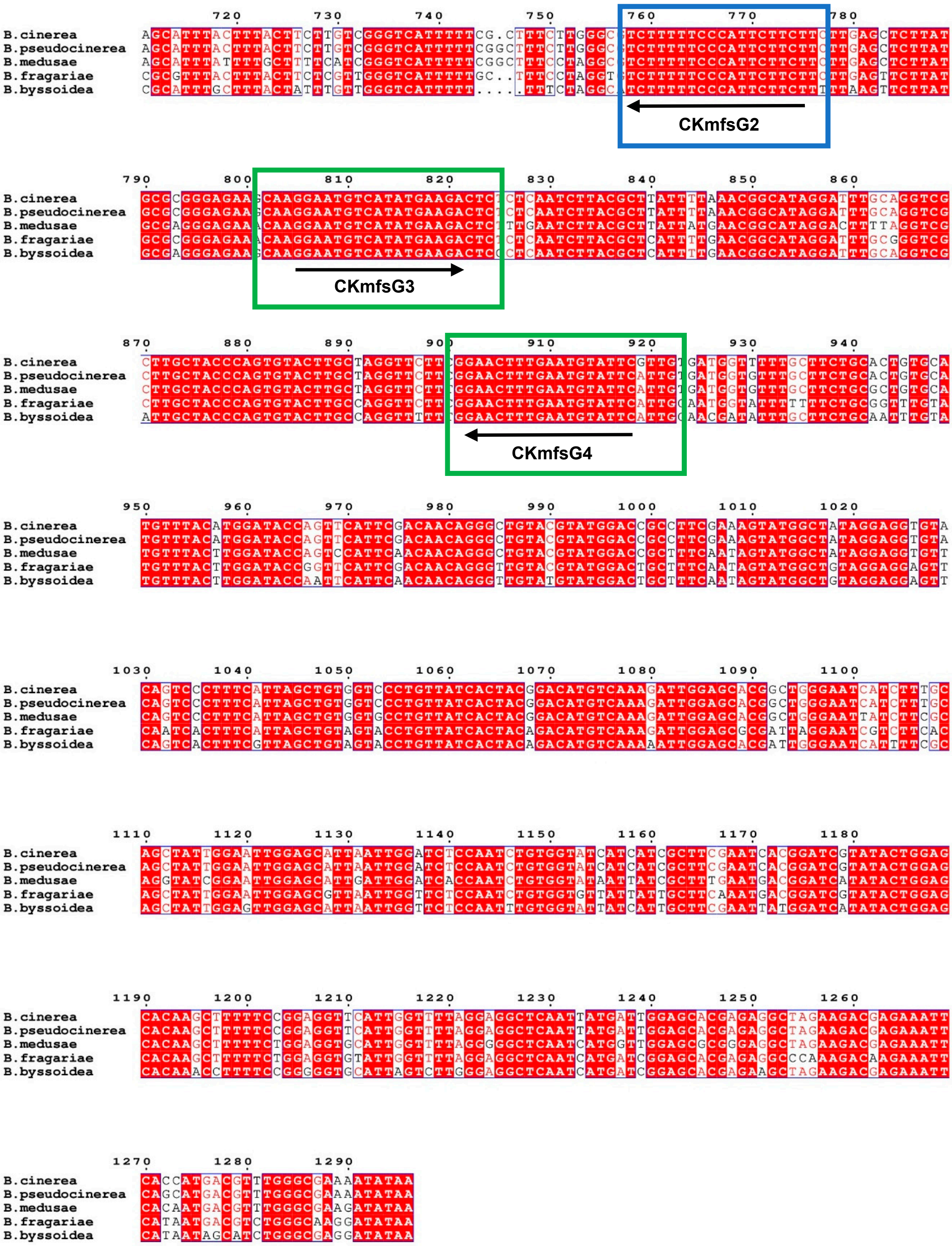

Supplementary Table S1. List of oligonucleotides used for the expression analysis study.

Name of the Oligo	Sequence	Description of the primer
CKmfsG1	GTTATGGGAATAGCG	Identification of the *mfsG* gene in other *Botrytis* species
CKmfsG2	AGAAGAATGGGAAAAAGA	Identification of the *mfsG* gene in other *Botrytis* species
CKmfsG3	GGAATGTCATATGAAGA	Identification of the *mfsG* gene in other *Botrytis* species
CKmfsG4	GAATACATTCAAAGTTC	Identification of the *mfsG* gene in other *Botrytis* species
FwMfsGcinerea	GTATACTGGAGCACAAGCTT	mRNA level quantification of the gene *mfsG* in *B. cinerea*
RvMfsGcinerea	GGTGAATTTCTCGTCTTCTAG	mRNA level quantification of the gene *mfsG* in *B. cinerea*
FwMfsGpseudocinerea	ATACTGGAGCACAAGCTTT	mRNA level quantification of the gene *mfsG* in *B. pseudocinerea*
RvMfsGpseudocinerea	CTGAATTTCTCGTCTTCTAGC	mRNA level quantification of the gene *mfsG* in *B. pseudocinerea*
FwMfsGbyssoidea	TTAGTCTTGGGAGGCTCAAT	mRNA level quantification of the gene *mfsG* in *B. byssoidea*
RvMfsGbyssoidea	TTATATCCTCGCCCAGATG	mRNA level quantification of the gene *mfsG* in *B. byssoidea*
FwActAcinerea	TTTGAGACCTTCAACGCCCC	mRNA level quantification of the gene *BcactA* in *B. cinerea*
RvActAcinerea	ACGTGAGTAACTCCGTCACC	mRNA level quantification of the gene *BcactA* in *B. cinerea*
FwActApseudocinerea	GGTGGTATCCACGTCACTA	mRNA level quantification of the gene *BcactA* in *B. pseudocinerea*
RvActApseudocinerea	CATAACAATGTTACCATACAAATC	mRNA level quantification of the gene *BcactA* in *B. pseudocinerea*
FwActAbyssoidea	TGACGATGCTCCAAGAGC	mRNA level quantification of the gene *BcactA* in *B. byssoidea*
RvActAbyssoidea	ATTGTCAATGACGAGGGCT	mRNA level quantification of the gene *BcactA* in *B. byssoidea*
Fwtubcinerea	TCCTTTCGGTCAACTCTTCCG	mRNA level quantification of the gene β-tubuline in *B. cinerea*
Rvtubcinerea	CACCCTCAGTGTTAATGACCC	mRNA level quantification of the gene β-tubuline in *B. cinerea*
Fwtubpseudocinerea	GATCTCCAACTTGAGCGTA	mRNA level quantification of the gene β-tubuline in *B. pseudocinerea*
Rvtubpseudocinerea	TGCTCGCCAGAGATAGTTT	mRNA level quantification of the gene β-tubuline in *B. pseudocinerea*
Fwtubbyssoidea	GGTGATCAATTCACTGCTAT	mRNA level quantification of the gene β-tubuline in *B. byssoidea*
Rvtubbyssoidea	TTCATGTTGGACTCAGCCT	mRNA level quantification of the gene β-tubuline in *B. byssoidea*
